# Blood pool activity on F-18 FDG PET/CT as a possible imaging biomarker of metabolic syndrome

**DOI:** 10.1038/s41598-020-74443-9

**Published:** 2020-10-15

**Authors:** Ji-In Bang, Chang Mo Moon, Hye Ok Kim, Seo Young Kang, Hai-Jeon Yoon, Bom Sahn Kim

**Affiliations:** 1 Department of Nuclear Medicine, CHA Bundang Medical Center, CHA University, Seongnam, South Korea; 2 Depratment of Internal Medicine, Ewha Womans University School of Medicine, Seoul, South Korea; 3 Department of Nuclear Medicine, Ewha Womans University School of Medicine, Seoul, South Korea

**Keywords:** Endocrinology, Molecular medicine

## Abstract

Association of blood pool (BP) and adipose tissue activity from F-18 fluorodeoxyglucose positron-emission tomography/computed tomography (FDG PET/CT) with the parameters of metabolic syndrome (MetS) and different MetS/obesity types were investigated. 245 subjects underwent FDG PET/CT scan for health check-ups were investigated retrospectively. Associations of BP (BP SUV: SUV_max_, SUV_mean_), visceral (VAT SUV), and subcutaneous adipose tissue (SAT SUV) activity with parameters of MetS, body mass index (BMI), and lipid profiles were analyzed. MetS/obesity types were subdivided into metabolically healthy obese (MHO) and metabolically unhealthy obese (MUO). BP SUV was higher in subjects with MetS (t-test, P < 0.005), and was associated with MetS from multivariable binary logistic regression (OR 5.232 P = 0.010). BP SUV was statistically higher in MUO than in MHO (P < 0.05) along with blood pressure, triglycerides, and HDL-cholesterol. Multivariable binary logistic regression analysis showed MUO had higher blood pressure and BP SUV, while lower HDL-cholesterol relative to MHO after adjusting for triglycerides.

## Introduction

Metabolic syndrome (MetS) is one of the most serious health conditions worldwide, which is associated with increased cardiocerebrovascular disease and all-cause mortality^1^. Although the definition of MetS is controversial and has been changed, its main components are central obesity, dyslipidemia, elevated arterial blood pressure, and dysregulated glucose homeostasis or insulin resistance^2^. The main factor of its pathophysiology is focused on central obesity, which is a marker of dysfunctional adipose tissues inducing insulin resistance and systemic inflammation, thus resulting in the final presentation of several diseases^3^. It is crucial to identify and assess the individuals prone to MetS for intervening with the pathologic course.

Interestingly, a unique population of obese individuals has been defined as “metabolically healthy, but obese (MHO)”; these individuals show a healthy metabolic profile despite having phenotypes of obesity^4,5^. Several studies have shown that mortality risk in MHO individuals is comparable to that of “metabolically unhealthy and obese (MUO)” individuals and that a significant portion of MHO eventually develop metabolic abnormalities^6–10^. Therefore, understanding and characterizing features that can enhance the aggravating effect of obesity to metabolic health is important.

F-18 fluorodeoxyglucose (FDG) positron emission tomography/computed tomography (PET/CT) has been the main tool for tumor imaging in oncology. However, due to the ability to represent the physiologic metabolic state of the entire body in vivo, there have been several attempts to use FDG PET/CT for non-tumor imaging biomarkers. For example, measuring the adipose tissue metabolism using FDG PET/CT has been attempted to represent the functional state of adipose tissues^11–13^. In addition, the metabolic activity of organs, such as blood pool, liver, and spleen, has been investigated with several other diseases related to systemic inflammation or metabolic state^14,15^. Among these, the blood pool activity has been considered as a reference organ which could be used to correct or normalize the metabolic activity across the oncologic imaging^16^. However, the blood pool activity could differ with metabolic states^17^. Therefore, instead of being a reference organ, the blood pool activity itself could be the possible parameter reflecting the metabolic status.

In this study, we investigated the metabolic activity of adipose tissue and blood pool using FDG PET/CT with parameters related to metabolic syndrome and obesity status to evaluate the possible imaging biomarkers for MetS.

## Materials and methods

### Study subjects

The institutional review board of Ewha Womans University Mokdong Hospital approved this study and exempted the need for individual informed consent (IRB no. 2019-12-017). All research was performed in accordance with the principles of the 1975 Declaration of Helsinki (2013 revision). We retrospectively reviewed the FDG PET/CT examination results of health check-ups of adults between January 2011 and February 2015. We excluded subjects who were non-Koreans, had a history of malignancy and/or autoimmune disease, and had insufficient data to determine metabolic syndrome using the modified National Cholesterol Education Program-Adult Treatment Panel (NCEP-ATP) III criteria for Koreans^18^. In addition, we excluded subjects who were previously diagnosed with diabetes or were treated with diabetes medication at the time of FDG PET/CT examination to eliminate the possible confounding effects.

Metabolic syndrome was confirmed when three or more modified NCEP-ATP III criteria for Koreans were met^18^. For classification of obesity, body mass index (BMI, in kg/m^2^) cut-offs were used with lean < 23 and obese ≥ 23^19^.

### Data collection

Data included age at the time of FDG PET/CT examination, sex, body weight, height, waist circumference, BMI (defined as the ratio of body weight in kilograms to the square of height in meters), systolic blood pressure (SBP), diastolic blood pressure (DBP), fasting serum glucose level, and serum lipid test results (triglycerides, total, low-density lipoprotein, and high-density lipoprotein–cholesterol); these data were collected from electronic medical records. All data were measured on the same day of FDG PET/CT examination or within 12 months of imaging.

### Acquisition of F-18 FDG PET/CT

The acquisition of FDG PET/CT was done as previously described in detail^20^. All subjects fasted for at least 6 h (11.4 ± 1.6 h) before the FDG PET/CT scan, and their blood glucose level was measured before intravenous injection and confirmed to be less than 200 mg/dL (89.8 ± 12.0 mg/dL). Each subject was injected with 5.18 MBq/kg of F-18 FDG. A non-contrast CT scan at 120 kVp was performed for attenuation correction and obtaining anatomical information. A PET scan covering the skull base to the upper thigh was acquired using a Siemens Biograph mCT with 128-slice CT (Siemens Medical Solutions, Erlangen, Germany) at 1 h (65.2 ± 7.7 min) after FDG injection. Three-dimensional emission was used for the acquisition parameters with 2-min scanning per bed position. The spatial resolution at the center of PET was 2.0 mm full width at half maximum (FWHM) in the transaxial direction. PET images were reconstructed to 200 × 200 matrices and 3.4 mm × 3.4 mm pixel sizes with a 3.0-mm slice thickness using a three-dimensional OSEM iterative algorithm (2 iterations and 21 subsets) with time-of-flight and point spread functions.

### Measurement of fat and blood pool metabolism

All PET/CT data were analyzed using commercially available systems (Syngo.via; Siemens Medical Solutions, Erlangen, Germany). The standardized uptake value (SUV) was corrected for the individual patient body weight, and a board-certified nuclear medicine physician reviewed the images and measurements. The maximum or mean SUV of blood pool (BP SUV_max_, BP SUV_mean_) was measured using the spherical volume of interest drawn manually on the center of the ascending aorta, not including the metabolic activity of the aortic wall itself. The measurements of the metabolic activity of visceral adipose tissue (VAT) and subcutaneous adipose tissue (SAT) were acquired as previously described in detail with minor modification^12,20–22^. In brief, adipose tissue areas were identified using predefined Hounsfield units (HU, range − 190 to − 30) from CT images^23^. To measure VAT metabolism, region of interests (ROIs) (7–15 mm) were located on five slices of abdominal VAT below the kidneys. The placement of ROIs was carefully reviewed to avoid possible spillover from neighboring organs. SUV of each ROI was retrieved, and five VAT SUV values from a subject were averaged to represent the maximum or mean VAT metabolism (VAT SUV_max_, VAT SUV_mean_). To measure SAT metabolism, ROIs (7–15 mm) were located on five slices of the abdominal subcutaneous area around the umbilical region. SUV of each ROI was retrieved, and five SAT SUV values from a subject were averaged to represent the maximum or mean SAT metabolism (SAT SUV_max_, SAT SUV_mean_).

### Statistical analysis

Clinical parameters for determining MetS or possible risk factors for MetS and VAT SUV, SAT SUV and BP SUV were compared using independent t-test according to the presence of MetS. To identify the parameters associated with MetS status, multivariable binary logistic regression was performed. To evaluate the relationships between BP SUV and other PET and clinical parameters among adipose tissue, blood pool activity, and clinical parameters for MetS, Pearson’s correlation analyses were employed. Comparison between metabolically healthy vs. unhealthy in obese subgroup was performed using independent t-test. A multivariable binary logistic regression analysis was performed for those with a *P* value less than 0.05 from univariate analysis. Receiver operating characteristic (ROC) curve analysis was used to determine the optimal cut-off value of FDG PET and clinical parameters to differentiate between MHO and MUO groups. All statistical analyses were performed using a commercial statistical software package (SPSS Ver. 19.0; SPSS Inc., Chicago, IL) and R statistical software (version 3.2.2, The R Foundation for Statistical Computing)^24^, and two-sided P-values < 0.05 were considered statistically significant.

## Results

### Patient characteristics

**Figure 1:**
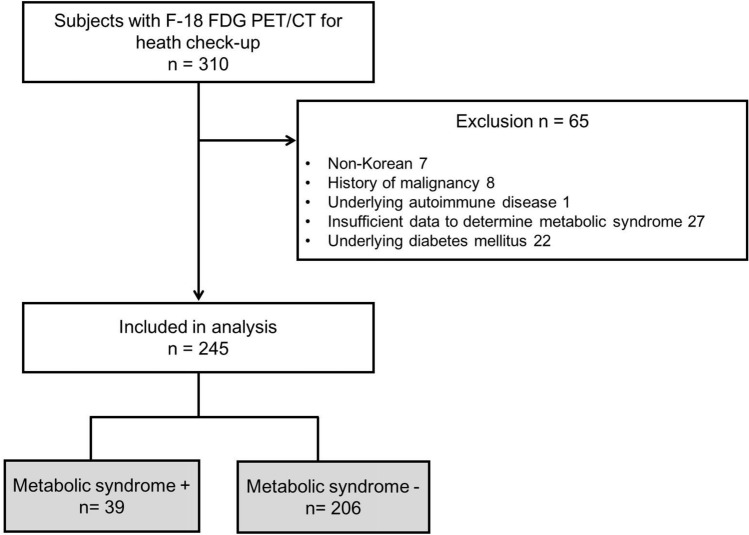
Study design and flow chart of subject selection.

We initially enrolled 310 subjects, and the following 65 subjects were excluded: non-Koreans (n = 7), those with a history of malignancy (n = 8), those with an underlying autoimmune disease (n = 1), those with insufficient data to determine metabolic syndrome (n = 27), and those with underlying diabetes mellitus (n = 22) (Fig. 1).

The final analysis included 245 subjects. Table 1 summarizes the demographics of 245 enrolled subjects. Among the study subjects, 39 subjects satisfied the criteria of metabolic syndrome.

**Table 1:** Characteristics of study subjects.

Characteristics	Total 245 subjects
Age, years (range)	51.8 ± 7.7 (35–71)
**Gender (number, %)**	
Female	82 (33.5%)
Male	163 (66.5%)
BMI (kg/m^2^)	24.3 ± 2.9 (17.5–34.2)
Lean (number, %)	85 (34.7%)
Obese (number, %)	160 (65.3%)
Central obesity (number, %)	83 (33.9%)
Hypertriglyceridemia (number, %)	48 (19.6%)
Low HDL-cholesterol (number, %)	50 (20.4%)
High blood pressure (number, %)	98 (40.0%)
High fasting glucose level (number, %)	35 (14.3%)
Metabolic syndrome (number, %)	39 (15.9%)

### Comparison of clinical and PET metabolic parameters according to the presence of metabolic syndrome

All clinical parameters constituting of the NCEP-ATP III criteria were significantly different according to the presence of MetS. Besides, BMI, HbA1c, total cholesterol, and BP SUV were significantly higher in the group with MetS compared with group without MetS (Table 2). However, VAT SUV and SAT SUV was not significantly different between the two groups.

**Table 2:** Comparison of clinical and PET-derived parameters according to the presence of metabolic syndrome.

	With metabolic syndrome (n = 39)	Without metabolic syndrome (n = 206)	*P* value
**Waist circumference (cm)**			< 0.001
For female (n = 82)	90.6 ± 5.9	75.0 ± 6.7	< 0.001
For male (n = 163)	96.0 ± 8.7	85.4 ± 6.7	< 0.001
SBP (mmHg)	131.2 ± 9.1	121.0 ± 12.7	< 0.001
DBP (mmHg)	79.9 ± 7.1	72.8 ± 8.8	< 0.001
Triglycerides (mg/dL)	198.4 ± 118.5	106.3 ± 82.5	< 0.001
**HDL-cholesterol (mg/dL)**			
For female (n = 82)	44.6 ± 9.8	60.3 ± 13.9	< 0.001
For male (n = 163)	45.9 ± 9.6	52.7 ± 10.9	< 0.001
Fasting glucose (mg/dL)	95.0 ± 16.8	88.9 ± 10.7	0.033
BMI (kg/m^2^)	27.5 ± 2.7	23.6 ± 2.6	< 0.001
HbA1c (%)	6.1 ± 0.4 (n = 37)	5.8 ± 0.5 (n = 190)	0.010
Total cholesterol (mg/dL)	203.4 ± 38.3	190.3 ± 32.9	0.027
LDL-cholesterol (mg/dL)	129.3 ± 40.5	120.5 ± 31.6	0.132
BP SUV_max_	2.24 ± 0.32	2.07 ± 0.29	0.002
BP SUV_mean_	1.95 ± 0.28	1.82 ± 0.25	0.004
VAT SUV_max_	1.31 ± 0.83	1.52 ± 0.83	0.165
VAT SUV_mean_	0.55 ± 0.26	0.58 ± 0.28	0.515
SAT SUV_max_	0.36 ± 0.15	0.35 ± 0.13	0.673
SAT SUV_mean_	0.24 ± 0.08	0.23 ± 0.62	0.409

*SBP* systolic blood pressure, *DBP* diastolic blood pressure, *BP* blood pool, *VAT* visceral adipose tissue, *SAT* subcutaneous adipose tissue.

Multivariable binary logistic regression was performed with parameters showed significantly different between groups with and without MetS, except the parameters which are constituted the NCEP-ATP III criteria. Although BMI is not a component of the NCEP-ATP III criteria, it has a strong correlation with waist circumference (*r* = 0.826). Multivariate model including BMI, HbA1c, and total cholesterol (Model 1) showed that BMI and total cholesterol were significant independent variables for MetS. Model 2 including BP SUV instead of BMI showed that BP SUV, HbA1c, and total cholesterol were significant independent variables for MetS. (Table 3).

**Table 3:** Multivariable binary logistic regression to identify independent parameters associated with metabolic syndrome status.

	Variable	*P* value	Odds ratio	95% CI
Model 1	BMI (kg/m^2^)	< 0.001	1.668	1.400–1.989
	Total cholesterol (mg/dL)	0.025	1.015	1.002–1.028
	Hba1c (%)	0.234	1.463	0.782–2.736
Model 2	BP SUV_max_	0.010	5.232	1.490–18.369
	Total cholesterol (mg/dL)	0.032	1.012	1.001–1.023
	Hba1c (%)	0.048	1.821	1.006–3.298

### Relationships between blood pool activity and clinical parameters

Table 4 shows the correlations between BP SUV and other PET and clinical parameters including MetS components. BP SUV was positively correlated with waist circumference, DBP, fasting glucose, and triglyceride level. It was also positively correlated with BMI, HbA1c, total cholesterol level, VAT SUV, and SAT SUV from Pearson correlation analyses.

**Table 4:** Relationships between blood pool activity (BP SUV) and clinical parameters for metabolic syndrome in simple correlation analyses.

	BP SUV_max_		BP SUV_mean_	
	Correlation coefficient, r	*P* value	Correlation coefficient, r	*P* value
BMI (kg/m^2^)	0.406	< 0.001	0.359	< 0.001
Waist circumference (cm)	0.378	< 0.001	0.315	< 0.001
SBP (mmHg)	0.067	0.294	0.082	0.204
DBP (mmHg)	0.139	0.030	0.149	0.020
Fasting glucose (mg/dL)	0.212	0.001	0.187	0.003
Triglycerides (mg/dL)	0.228	0.001	0.261	0.002
HDL-cholesterol (mg/dL)	− 0.046	0.478	− 0.108	0.230
Hba1c (%)	0.148	0.026	0.186	0.107
Total cholesterol (mg/dL)	0.147	0.021	0.110	0.085
LDL-cholesterol (mg/dL)	0.081	0.205	0.035	0.588
VAT SUV_max_	0.325	< 0.001	0.254	< 0.001
VAT SUV_mean_	0.255	< 0.001	0.221	< 0.001
SAT SUV_max_	0.321	< 0.001	0.280	< 0.001
SAT SUV_mean_	0.215	0.001	0.211	0.001

*SBP* systolic blood pressure, *DBP* diastolic blood pressure, *VAT* visceral adipose tissue, *SAT* subcutaneous adipose tissue.

### Comparison of clinical and PET metabolic parameters between metabolically healthy obese and metabolically unhealthy obese group

Study subjects were divided according to BMI and metabolic status: metabolically healthy lean (MHL, healthy metabolic status and BMI is below 23, n = 68), metabolically healthy obese (MHO, healthy metabolic status and BMI is above 23, n = 138), metabolically unhealthy lean (MUL, unhealthy metabolic status and BMI is below 23, n = 0), and metabolically unhealthy obese (MUO, unhealthy metabolic status and BMI is above 23, n = 39). For non-obese subjects (BMI < 23), comparison between MHL and MUL could not be performed because there was no subject classified as MUL group. Regarding obese subjects (BMI ≥ 23), clinical and PET metabolic parameters were compared between MHO and MUO group. Comparison analysis showed that SBP, DBP, triglyceride, HDL-cholesterol levels, BP SUV_max_, and BP SUV_mean_ were significantly different between the two groups (Table 5).

**Table 5:** Comparison of clinical and PET-derived parameters according to BMI and metabolic status.

	BMI < 23, Non-obese		BMI ≥ 23, Obese		
	MHL (n = 68)	MUL (n = 0)	MHO (n = 138)	MUO (n = 39)	*P* value
SBP (mmHg)	115.4 ± 12.5	–	123.7 ± 11.8	131.2 ± 9.0	< 0.001
DBP (mmHg)	67.7 ± 7.5	–	75.3 ± 8.2	79.9 ± 7.1	0.002
Triglycerides (mg/dL)	81.3 ± 33.8	–	118.5 ± 95.6	198.3 ± 118.5	< 0.001
HDL-cholesterol (mg/dL)	59.9 ± 15.0	–	52.8 ± 10.2	45.4 ± 9.5	< 0.001
Fasting glucose (mg/dL)	87.8 ± 8.8	–	89.3 ± 11.5	95.0 ± 16.7	0.055
Hba1c (%)	5.7 ± 4.1	–	5.8 ± 0.5	6.0 ± 0.7	0.101
Total cholesterol (mg/dL)	190.6 ± 31.7	–	190.0 ± 33.6	203.4 ± 38.2	0.053
LDL-cholesterol (mg/dL)	117.7 ± 29.2	–	121.8 ± 32.7	129.2 ± 40.5	0.24
BP SUV_max_	1.96 ± 0.28	–	2.12 ± 0.27	2.26 ± 0.31	0.035
BP SUV_mean_	1.74 ± 0.25	–	1.85 ± 0.24	1.94 ± 0.27	0.041
VAT SUV_max_	1.29 ± 0.76	–	1.32 ± 0.86	1.51 ± 0.82	0.243
VAT SUV_mean_	0.60 ± 0.24	–	0.52 ± 0.25	0.58 ± 0.28	0.209
SAT SUV_max_	0.36 ± 0.12	–	0.36 ± 0.15	0.35 ± 0.13	0.834
SAT SUV_mean_	0.25 ± 0.08	–	0.23 ± 0.07	0.22 ± 0.06	0.714

*MHL* metabolic healthy lean, *MUL* metabolic unhealthy lean, *MHO* metabolic healthy obese, *MUO* metabolic unhealthy obese, *BP* blood pool, *SBP* systolic blood pressure, *DBP* diastolic blood pressure, *VAT* visceral adipose tissue, *SAT* subcutaneous adipose tissue.

Binary logistic regression analysis showed that SBP, HDL-cholesterol, and BP SUV were significantly associated metabolically unhealthy after adjusting for triglyceride level (Table 6).

**Table 6:** Results of multivariate binary logistic regression comparing MHO vs. MUO groups.

	P value	Odds ratio	95% CI
SBP (mmHg)	< 0.001	1.082	1.037–1.129
Triglyceride (mg/dL)	0.076	1.004	1.000–1.008
HDL-cholesterol (mg/dL)	0.001	0.919	0.873–0.967
BP SUV_max_	0.033	5.002	1.137–22.011

*SBP* systolic blood pressure, *MHO* metabolic healthy obese, *MUO* metabolic unhealthy obese, *BP* blood pool, *SBP* systolic blood pressure.

### Predictive performances of clinical and PET metabolic parameters for MUO

**Figure 2:**
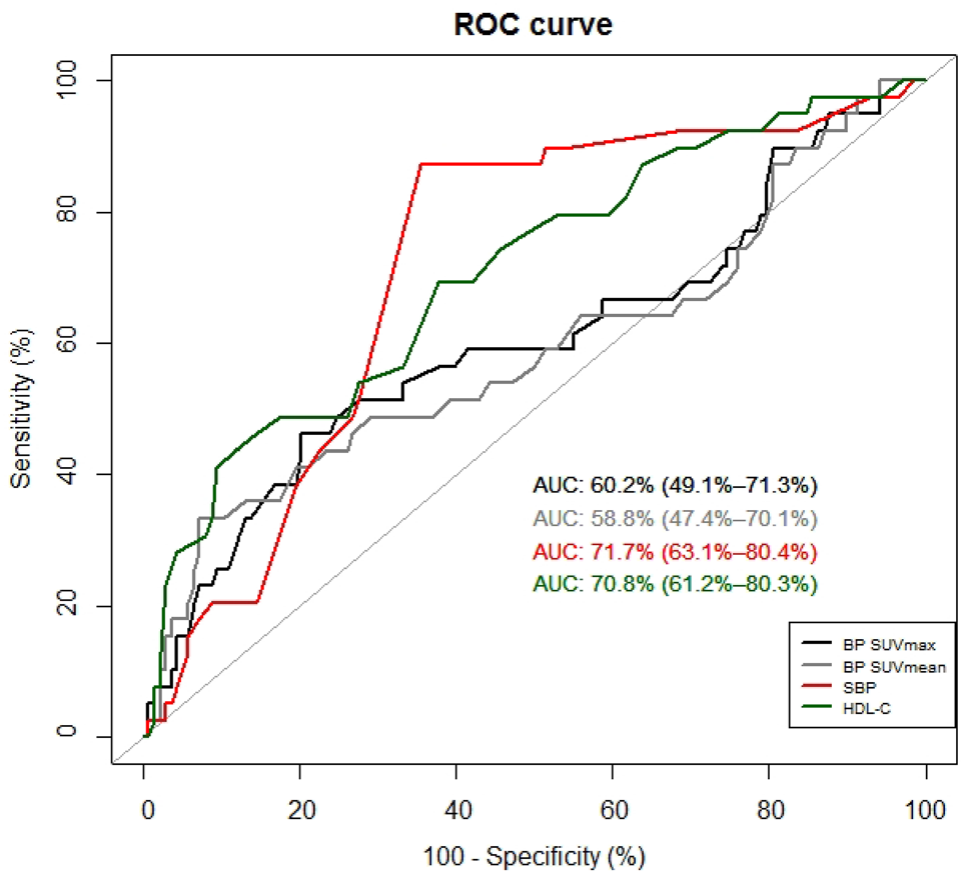
ROC curves for differentiating the MUO from MHO by BP SUV, SBP, and HDL-cholesterol level.

From ROC analysis to differentiate the MUO from MHO, respective optimal cut-off values for SBP, HDL-cholesterol level, and BP SUV were 129 mmHg (area under the curve 0.717, sensitivity 87.2%, specificity 64.5%), 41 mg/dL (area under the curve 0.708, sensitivity 43.6%, specificity 88.4%), and 2.35 (area under the curve 0.602, sensitivity 46.2%, specificity 79.7%; Fig. 2). Comparison of ROC curves showed that no significant differences between areas under curve (BP SUV vs. SBP, *p* = 0.135; BP SUV vs. HDL-cholesterol, *p* = 0.207; SBP vs. HDL-cholesterol, *p* = 0.894).

## Discussion

In this study, blood pool activity was significantly higher in subjects with metabolic syndrome and was significantly correlated with risk factors of metabolic syndrome. Considering obese subjects with BMI ≥ 23, blood pool activity was significantly higher in the MUO group compared with MHO group. Adipose tissue metabolic activities were correlated with blood pool activity; however, no adipose tissue metabolic activity was significantly different among different metabolic and obesity groups.

Blood pool is commonly used as the background in FDG PET/CT scans due to its easy and reproducible measurements in oncology^16,25,26^. However, the interaction of the biodistribution of FDG in normal tissues or tumors is complex^27^. Previous studies have shown that metabolic burden from oncologic patients could change the biodistribution of FDG, and therefore, the metabolic activity of reference organs, such as the liver or blood pool, could be affected^28^. In case of FDG PET/CT on healthy subjects, blood pool activity may be the overall result of the metabolism of the whole body, which would be closely related to the metabolic state. Insulin resistance, which is caused by central obesity, occurs in target organs, such as adipose tissue and muscles^29^. Insulin resistance leads to impeded glucose uptake in muscles through glucose transporter, which is also the transporter for FDG. Therefore, a state of insulin resistance may affect the FDG biodistribution of major organs and result in elevated blood pool activity. From our results, BP SUV was significantly higher in the group with metabolic syndrome. BMI strongly correlate with the waist circumference, which is a component of the NCEP-ATP criteria, and thus is a known predictor of MetS. We performed multivariable binary logistic regression analysis by using two different statistical model including BP SUV and BMI, respectively, and showed that BP SUV is also a significant indicator comparable to BMI. Interestingly, when we analyzed the subgroups divided by metabolic states and BMI in detail, BP SUV showed different distribution among MHO and MUO groups. Multivariable binary logistic regression analysis demonstrated that BP SUV was an independent parameter of MUO along with being a composite parameter of the NCEP-ATP III criteria. Moreover, ROC analysis to differentiate MUO from MHO suggested no significant difference of predictive performance of BP SUV compared to other composite parameters. Although this study had limitation due to the cross-sectional retrospective design, the blood pool activity from FDG PET/CT may be a possible imaging biomarker for metabolic unhealthy, and future study will be needed with longitudinal observational study including the conversion of MHO to MUO. In addition, using blood pool activity as reference value for FDG PET/CT may be invalid, particularly for non-oncologic, healthy subjects.

Metabolic activity of adipose tissue has been assessed to represent the possible evidence of dysfunctional adipocytes using FDG PET/CT^12,13^. Oliveira et al. showed that metabolic activity of visceral adipose tissue in MHO individuals using FDG PET/CT was similar to that of MUO individuals while being significantly lower than that of MHL individuals, and this may have been due to dysfunctional adipocytes^12^. However, in other studies, the metabolic activity of adipose tissue was positively correlated with inflammatory state or metabolic risk factors^13^. These opposite results could be due to methodological differences in using the ratio value with blood pool activity. In our study, blood pool activity itself showed a significant association with metabolic syndrome or its risk factors. Therefore, blood pool activity may reflect the pathological state of the whole body. Although the metabolic activity of adipose tissue was not significantly different between normal subjects and metabolic syndrome subjects, it was positively correlated with blood pool activity, and there was a tendency that VAT SUV was higher in MHO group than in the MUO group in our study.

Our study has several limitations. First, this is not a longitudinal observational study. Therefore, future studies should evaluate the meaning of BP SUV on whether it predicts the conversion of MHO to MUO or worse outcome of MetS. Second, inflammatory markers, such as ESR or CRP, were not included because only a limited number of subjects were included. Including inflammatory biomarkers would have been more useful to evaluate the MetS and chronic inflammation. In addition, we only enrolled Korean subjects who underwent FDG PET/CT for health check-up; therefore, the findings might be biased and not representative of the general population. Above all, as this study used the semi quantification of FDG PET with SUV value, the absolute quantification in whole body glucose metabolism should be needed for absolute linkage in physiologic process.

## Conclusion

Blood pool activity from FDG PET/CT is significantly different among different metabolic syndrome states. Blood pool FDG activity could be a possible imaging biomarker of metabolic syndrome, particularly for differentiating between MHO and MUO subjects. In addition, blood pool FDG activity may not be a valid reference value for PET/CT imaging in oncology because it depends on the metabolic state of the patient.

## References

[1] Engin A (2017). The definition and prevalence of obesity and metabolic syndrome. Adv. Exp. Med. Biol..

[2] Kassi E, Pervanidou P, Kaltsas G, Chrousos G (2011). Metabolic syndrome: Definitions and controversies. BMC Med..

[3] Després J-P, Lemieux I (2006). Abdominal obesity and metabolic syndrome. Nature.

[4] Karelis AD, St-Pierre DH, Conus F, Rabasa-Lhoret R, Poehlman ET (2004). Metabolic and body composition factors in subgroups of obesity: What do we know?. J. Clin. Endocrinol. Metab..

[5] Sims EA (2001). Are there persons who are obese, but metabolically healthy?. Metab. Clin. Exp..

[6] Ärnlöv J, Ingelsson E, Sundström J, Lind L (2010). Impact of body mass index and the metabolic syndrome on the risk of cardiovascular disease and death in middle-aged men. Circulation.

[7] Hinnouho G-M, Czernichow S, Dugravot A, Batty GD, Kivimaki M, Singh-Manoux A (2013). Metabolically healthy obesity and risk of mortality: Does the definition of metabolic health matter?. Diabetes Care.

[8] Kouvari M, Panagiotakos DB, Yannakoulia M, Georgousopoulou E, Critselis E, Chrysohoou C (2019). Transition from metabolically benign to metabolically unhealthy obesity and 10-year cardiovascular disease incidence: The ATTICA cohort study. Metab. Clin. Exp..

[9] Kuk JL, Ardern CI (2009). Are metabolically normal but obese individuals at lower risk for all-cause mortality?. Diabetes Care.

[10] Mongraw-Chaffin M, Foster MC, Anderson CAM, Burke GL, Haq N, Kalyani RR (2018). Metabolically healthy obesity, transition to metabolic syndrome, and cardiovascular risk. J. Am. Coll. Cardiol..

[11] Christen T, Sheikine Y, Rocha VZ, Hurwitz S, Goldfine AB, Di Carli M (2010). Increased glucose uptake in visceral versus subcutaneous adipose tissue revealed by PET imaging. JACC Cardiovasc. Imaging..

[12] Oliveira AL, Azevedo DC, Bredella MA, Stanley TL, Torriani M (2015). Visceral and subcutaneous adipose tissue FDG uptake by PET/CT in metabolically healthy obese subjects. Obesity (Silver Spring, Md.).

[13] Tahara N, Yamagishi S-I, Kodama N, Tahara A, Honda A, Nitta Y (2015). Clinical and biochemical factors associated with area and metabolic activity in the visceral and subcutaneous adipose tissues by FDG-PET/CT. J. Clin. Endocrinol. Metab..

[14] Kim EJ, Kim S, Kang DO, Seo HS (2014). Metabolic activity of the spleen and bone marrow in patients with acute myocardial infarction evaluated by 18f-fluorodeoxyglucose positron emission tomograpic imaging. Circ. Cardiovasc. Imaging.

[15] Nam HY, Jun S, Pak K, Kim IJ (2017). Concurrent low brain and high liver uptake on FDG PET are associated with cardiovascular risk factors. Korean J. Radiol..

[16] Wahl RL, Jacene H, Kasamon Y, Lodge MA (2009). From RECIST to PERCIST: Evolving considerations for PET response criteria in solid tumors. J. Nucl. Med..

[17] Yoo ID, Lee SM, Lee JW, Oh MJ-E, Cho Y-J, Shin HS (2017). The influence of adipose tissue volume can significantly 18 affect the metabolic activity of reference organs in F-FDG PET/CT studies of a normal healthy population. Hell. J. Nucl. Med..

[18] Choi SH, Kim DJ, Lee KE, Kim YM, Song YD, Kim HD (2004). Cut-off value of waist circumference for metabolic syndrome patients in Korean adult population. J. Korean Soc. Study Obes..

[19] Seo MH, Lee WY, Kim SS, Kang JH, Kang JH, Kim KK (2019). 2018 Korean Society for the study of obesity guideline for the management of obesity in Korea. J. Obes. Metab. Syndr..

[20] Yoon HJ, Kim BS, Lee KE, Moon CM, Yoo J, Kim JS (2017). Glucose metabolism of visceral adipose tissue measured by 18F-FDG PET/CT is related to the presence of colonic adenoma. Medicine..

[21] Pahk K, Rhee S, Kim S, Choe JG (2016). Predictive role of functional visceral fat activity assessed by preoperative F-18 FDG PET/CT for regional lymph node or distant metastasis in patients with colorectal cancer. PLoS ONE.

[22] Vanfleteren LE, van Meerendonk AM, Franssen FM, Wouters EF, Mottaghy FM, van Kroonenburgh MJ (2014). A possible link between increased metabolic activity of fat tissue and aortic wall inflammation in subjects with COPD. A retrospective 18F-FDG-PET/CT pilot study. Respir. Med..

[23] Bucerius J, Vijgen GH, Brans B, Bouvy ND, Bauwens M, Rudd JH (2015). Impact of bariatric surgery on carotid artery inflammation and the metabolic activity in different adipose tissues. Medicine..

[24] R: A Language and Environment for Statistical Computing (R Foundation for Statistical Computing, 2015).

[25] Viglianti BL, Wong KK, Wimer SM, Parameswaran A, Nan B, Ky C (2017). Effect of hyperglycemia on brain and liver (18)F-FDG standardized uptake value (FDG SUV) measured by quantitative positron emission tomography (PET) imaging. Biomed. Pharmacother..

[26] Boktor RR, Walker G, Stacey R, Gledhill S, Pitman AG (2013). Reference range for intrapatient variability in blood-pool and liver SUV for 18F-FDG PET. J. Nucl. Med..

[27] Busing KA, Schonberg SO, Brade J, Wasser K (2013). Impact of blood glucose, diabetes, insulin, and obesity on standardized uptake values in tumors and healthy organs on 18F-FDG PET/CT. Nucl. Med. Biol..

[28] Viglianti BL, Wale DJ, Wong KK, Johnson TD, Ky C, Frey KA (2018). Effects of tumor burden on reference tissue standardized uptake for PET imaging: Modification of PERCIST criteria. Radiology.

[29] Zeyda M, Stulnig TM (2009). Obesity, inflammation, and insulin resistance—A mini-review. Gerontology..

